# Torsion of a normal ovary in the third trimester of pregnancy: a case report

**DOI:** 10.1186/1752-1947-2-378

**Published:** 2008-12-08

**Authors:** Arumugam Silja, Vaidyanathan Gowri

**Affiliations:** 1 Department of Obstetrics and Gynecology, Sultan Qaboos University Hospital, Red 2, Muscat, Oman; 2 College of Medicine, Sultan Qaboos University, Muscat, Oman

## Abstract

**Introduction:**

Adnexal torsion in advanced pregnancy is an uncommon emergency. Torsion usually occurs in ovaries with functional cysts or tumors. It is uncommon for a normal-sized ovary to undergo torsion in advanced gestation. We report torsion of a normal-sized ovary in the third trimester of pregnancy, most probably the first case report of its kind in the English-language literature.

**Case presentation:**

A 32-year-old Omani woman at 32-weeks gestation (gravida 2 para 1) was admitted with right iliac fossa pain, nausea and vomiting of 2 days duration, as well as a history of a similar episode one month earlier. On examination, a provisional diagnosis of appendicitis was made. Laparotomy revealed, however, that the right ovary was gangrenous and had undergone torsion.

**Conclusion:**

Adnexal torsion, though rare, should be kept in mind in the differential diagnosis of lower abdominal pain in advanced gestation. Although in our patient, the affected ovary could not be saved, an early diagnosis using imaging like Doppler of the adnexae will enable early intervention to save the ovaries of the patient, especially in young women.

## Introduction

Adnexal torsion is the fifth most common gynecological emergency with a reported incidence of 2.7% [1]. The incidence during pregnancy is one in 5000, occurring mostly in early pregnancy, especially following ovarian stimulation for the treatment of infertility [2]. The clinical symptoms of adnexal torsion in advanced pregnancy are non-specific and could be confused with other causes like appendicitis, cholecystitis and labor. This can lead to a delay in diagnosis and surgical management.

We report a case of torsion of a normal ovary during the third trimester of pregnancy.

## Case presentation

A 32-year-old Omani woman at 32-weeks gestation (gravida 2 para1; G2P1) was admitted with a history of right iliac fossa pain, nausea and vomiting of 2 days duration. She had no fever or urinary symptoms. She reported similar symptoms had occurred one month earlier, when she had presented at a different hospital and was given analgesics which relieved her of the symptoms until the time of the present admission.

On examination, the patient was afebrile and her vital signs were stable. Abdominal examination revealed a gravid uterus corresponding to 32 weeks with tenderness in the right lower quadrant. There was no uterine activity. An abdominal ultrasound scan revealed the fetal parameters corresponded to gestation with normal amniotic fluid and fetal activity. The non-stress test was reactive. An adnexal mass of 3.2 × 2.5 cm was discovered with internal echoes and irregular walls. Her hemoglobin was 11 g/dl and the white cell count was 10,500/mm^3^. The results of urine microscopy were normal.

The opinion of a general surgical team was sought and a provisional diagnosis of appendicitis was made. Laparotomy was conducted through a grid-iron incision. The appendix was normal in appearance. Minimal blood-stained peritoneal fluid was noted on opening the abdomen. The right ovary was gangrenous and had undergone torsion three times on its pedicle. Since there was no evidence of vascular supply on untwisting the ovary, it was unsalvageable and a salpingo-ovariectomy was performed. Histopathology confirmed a gangrenous ovary and fallopian tube.

The patient experienced an uneventful postoperative period. Pregnancy continued until 39 weeks and the patient vaginally delivered a healthy baby weighing 3200 g.

## Discussion

Adnexal torsion is rare in the second trimester of pregnancy and exceptional in the third trimester. Diagnosis is hampered by non-specific symptoms common in pregnancy. Early diagnosis is essential as it facilitates a conservative approach. When diagnosis is made early and the adnexa is hemorrhagic, simple detorsion is possible with good functional health [3]. The use of color Doppler appears to be promising in establishing the diagnosis [4]. However, a decreased blood flow should not rule out the suspicion of adnexal torsion. MRI is a potential alternative, as it can demonstrate signs of hemorrhagic infarction [5].

Laparoscopic management of a non-obstetric emergency in the third trimester of pregnancy has been reported to be feasible and safe by Upadhyay et al. [6]. Laparoscopic management needs skilled personnel and equipment. In our case, the grid-iron incision through McBurney's point was useful to explore the adnexa without uterine manipulation.

## Conclusion

An early diagnosis might have helped conserve our patient's ovary. Though rare, adnexal torsion should be considered in the differential diagnosis of acute abdominal pain in the third trimester of pregnancy.

## Consent

Written informed consent was obtained from the patient for publication of this case report and accompanying images. A copy of the written consent is available for review by the Editor-in-Chief of this journal.

## Competing interests

The authors declare that they have no competing interests.

## Authors' contributions

AS was involved in the management of the case and wrote the manuscript draft. VG conducted the literature research and revised the manuscript.
